# Modified internal limiting membrane flap technique for large chronic macular hole: Two case reports

**DOI:** 10.1097/MD.0000000000028412

**Published:** 2022-01-07

**Authors:** Keran Li, Yunfan Zhou, Weihua Yang, Qin Jiang, Xiangzhong Xu

**Affiliations:** Department of Fundus Disease, The Affiliated Eye Hospital of Nanjing Medical University, Nanjing, Jiangsu, China.

**Keywords:** autologous blood, case report, large chronic MH, modified ILM flap covering technique, sandwich-type

## Abstract

**Rationale:**

Internal limiting membrane (ILM) peeling and gas tamponade are the standardized treatments for macular holes (MHs). However, the close rate is low, and postoperative vision is unsatisfactory in large, chronic MHs. Currently, various modifications of the ILM flap techniques are being gradually applied for large MHs in the hope of obtaining better postoperative effects. This study described 2 successful cases achieved by “Sandwich-type” modified ILM flap covering technique in patients with large, chronic MHs.

**Patient concerns:**

A 62-year-old woman presented with decreased vision and visual distortion of the left eye for 18 months. Optical coherence tomography (OCT) showed the absence of full-thickness neuroepithelial tissue in the central fovea, with a minimum MH diameter of 742 μm and a base diameter of 1630 μm. A 57-year-old man experienced decreased visual acuity for 8 months. OCT showed the absence of full-thickness neuroepithelial tissue in the central fovea, with a minimum MH diameter of 713 μm and a basal diameter of 939 μm.

**Diagnoses:**

Two patients were diagnosed with large, chronic MH based on the OCT results and duration of the hole.

**Interventions:**

The 2 patients were treated with the “sandwich-type” modified ILM flap covering technique.

**Outcomes:**

Large, chronic MH closure was observed using SD-OCT, and the BCVA improved. The patients were very satisfied with the postoperative results.

**Lessons:**

“Sandwich-type” modified ILM flap covering technique may be a safe, effective way for large, chronic MH.

## 1 Introduction

A macular hole (MH) is a full-thickness or partial-thickness defect in the macular neuroepithelial layer. MH is divided into idiopathic and secondary MH according to its etiology. An idiopathic macular hole (IMH) accounts for approximately 80% of all MHs.^[1]^ The longitudinal traction caused by vitreous liquefaction and incomplete posterior vitreous detachment (PVD) to the macular retina and the tangential contraction of retinal glial cells that migrate on the vitreous/retinal boundary is speculated to be the 2 major factors in the formation and development of IMH.^[2,3]^ Secondary MH is common in patients with high myopia, eye trauma, or retinal surgery. The formation of secondary MH has been shown to be correlated with primary diseases, such as epiretinal membrane, macular edema, proliferative diabetic retinopathy (PDR), and rhegmatogenous retinal detachment (RRD).^[4]^ Pars plana vitrectomy (PPV) combined with internal limiting membrane (ILM) peeling is the conventional surgical treatment, which achieves 93% to 98% MH closure.^[5,6]^ However, this rate largely depends on the status of the fundus, base diameter, inner opening size, and chronicity. Previous studies have shown that for large MHs, the closure rate after conventional ILM peeling is low (55–80%). When the base diameter is >900 μm or is accompanied by high myopia, the closure rate is likely to decrease further.^[6,7]^ In addition, glial cells activated after peeling of the ILM are not only inefficient for the rearrangement or migration of photoreceptor cells and cause scar formation, but also lead to various complications, including retinal damage, thinning, edema, decreased contrast sensitivity, and macular displacement.^[8,9]^ Thus, surgical modification, especially for large chronic MH, has been the focus of intensive research in recent years. For instance, the inverted flap technique, ILM insertion, lens-capsule flap transplantation, free internal limiting membrane flap, and the pedicle internal limiting membrane transposition flap, heavy water, autologous blood, and hyaluronic acid gel are all being studied for the treatment of MH.^[10–12]^ However, an optimal method has not yet been identified. Thus, this study aimed to evaluate the anatomical and functional outcomes of 2 cases with large chronic MHs by applying a “sandwich-type modified ILM flap covering technique.”

## 2 Case presentation

The study protocol was approved by the Ethics Committees of the Affiliated Eye Hospital of Nanjing Medical University (2020011), and all participants provided written informed consent.

### 2.1 Case 1

**Figure 1:**
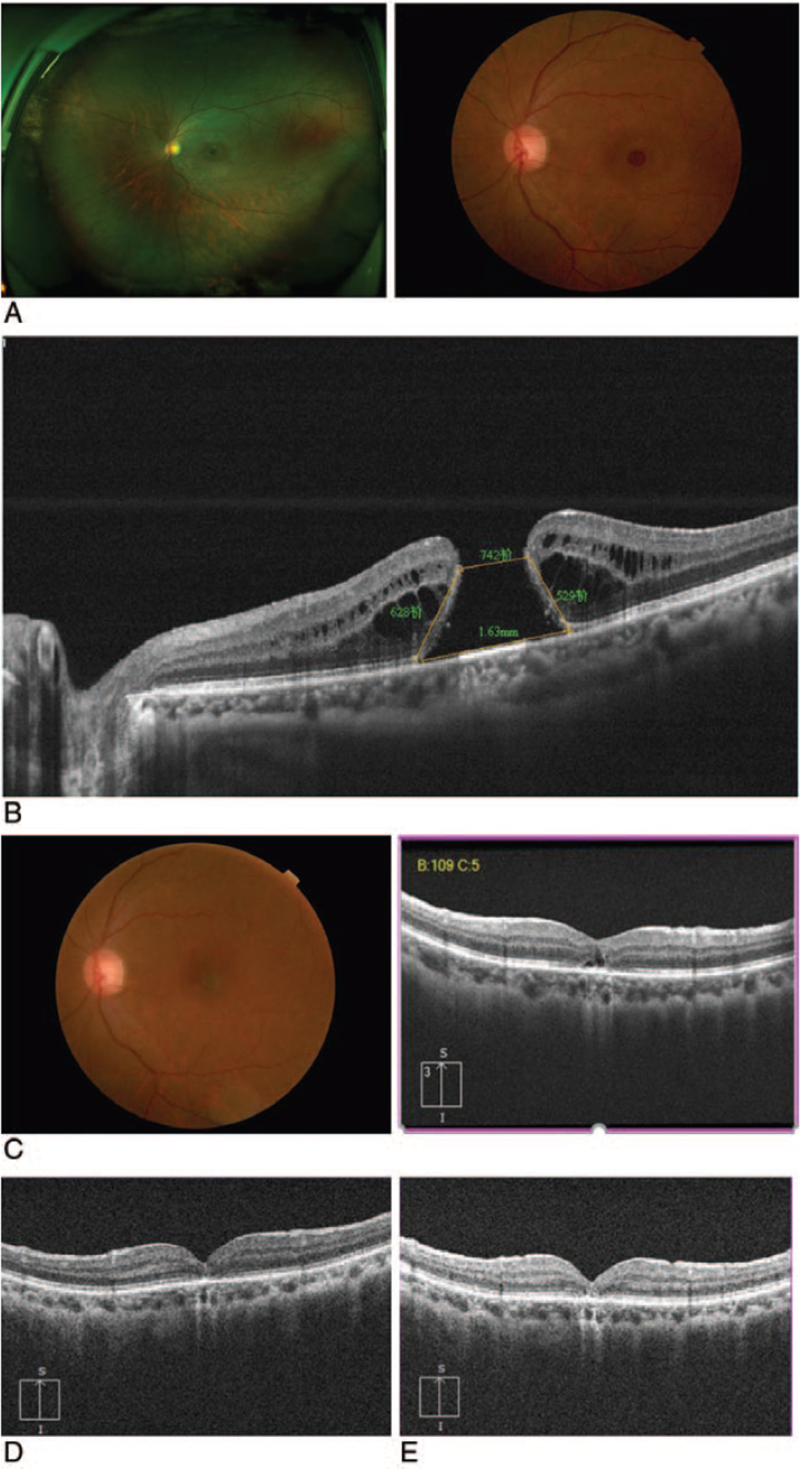
Case1 (A) The fundus image and wide-angle fundus image of the left eye. (B) Absence of full-thickness neuroepithelial tissue in the central fovea, with minimum MH diameter of 742 μm and base diameter of 1630 μm. (C): 1 month after surgery, the fundus image and OCT image demonstrated that the hole has closed, but the OCT showed the reflection of the external limiting membrane (ELM) and ellipsoid were discontinuous. (D,E) About 3 and 6 months after surgery, the OCT image showed ELM and ellipsoidal zone restored continuity.

**Figure 2:**
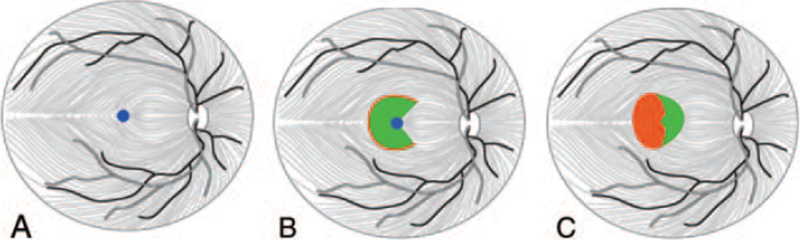
Modified inverted internal limiting membrane (ILM) flap covering technique; (A) Blue circle shows MH; (B) After autologous blood was dripped into the MH and ICG staining, A 270-degree C-shaped temporal ILM flap was peeled towards the hole but stopped at approximately 0.5 DD from the center of the fovea. ILM flap with a size of about 1.5 DD was trimmed; (C) The temporal flap was used to cover the hole, and then autologous blood was applied to fix the ILM flap to form a “Sandwich-type” structure.

A 62-year-old female suffered with decreased vision and visual distortion of the left eye for 18 months. She had no history of chronic diseases, such as hypertension or diabetes. However, RRD surgery was performed 24 months ago in the same eye, during which the retina was reattached, and the vitreous cavity was injected with silicone oil. Three months later, the silicone oil was removed by vitrectomy. Her best-corrected visual acuity (BCVA, LogMAR) was 0.22 in the right eye and 1.30 in the left eye, respectively. Intraocular pressure and anterior segments of both eyes were normal. Fundus examination showed no obvious abnormality in the right eye, while a full-thickness MH in the left eye and scar pigmentation secondary to laser were observed around the old retinal lesions (Fig. 1A). Spectral-domain optical coherence tomography (SD-OCT, Heidelberg Engineering, Germany) showed the absence of full-thickness neuroepithelial tissue in the central fovea, with a minimum MH diameter of 742 μm and a base diameter of 1630 μm. In addition, a small accumulation of intraretinal fluid in the perilesional area was observed (Fig. 1B). Due to the chronicity and the size of the MH as well as the history of RRD, the patient received “Sandwich-type” modified ILM flap covering technique of the left eye under general anesthesia in our hospital on June 4, 2020. Fresh autologous blood (0.3 mL) was obtained from the patient's antecubital vein, and 1 drop of autologous blood (approximately 0.1 mL) was injected gently into the MH.^[13]^ Indocyanine green (ICG) was used for 30 seconds to determine the shape of the internal limiting membrane. A 270°C temporal ILM flap was peeled towards the hole to fully release the tension, but we stopped at approximately 0.5 optic disc diameter (DD) from the center of the fovea. An ILM flap with approximately 1.5 D was trimmed, and the temporal flap was used to gently and carefully cover the hole without curl. Subsequently, a drop (about 0.1 mL) of autologous blood was applied to fix the ILM flap to form a sandwich structure (Fig. 2A--C). Air–fluid exchange was performed with gas tamponade with 15% perfluoropropane (C3F8) at the end of surgery. The patient was placed in the prone position after the operation (at least 80% of the time). At 30 days after the surgery, complete MH closure was observed by SD-OCT and fundus photography (Fig. 1C) and followed up for 3 to 6 months (Fig. 1D,E). The BCVA(LogMAR)improved from 1.30 to 0.70 within 3 months after the surgery, and the visual recovery remained unchanged after 6 months. The patient was extremely satisfied with the postoperative effects.

### 2.2 Case 2

**Figure 3:**
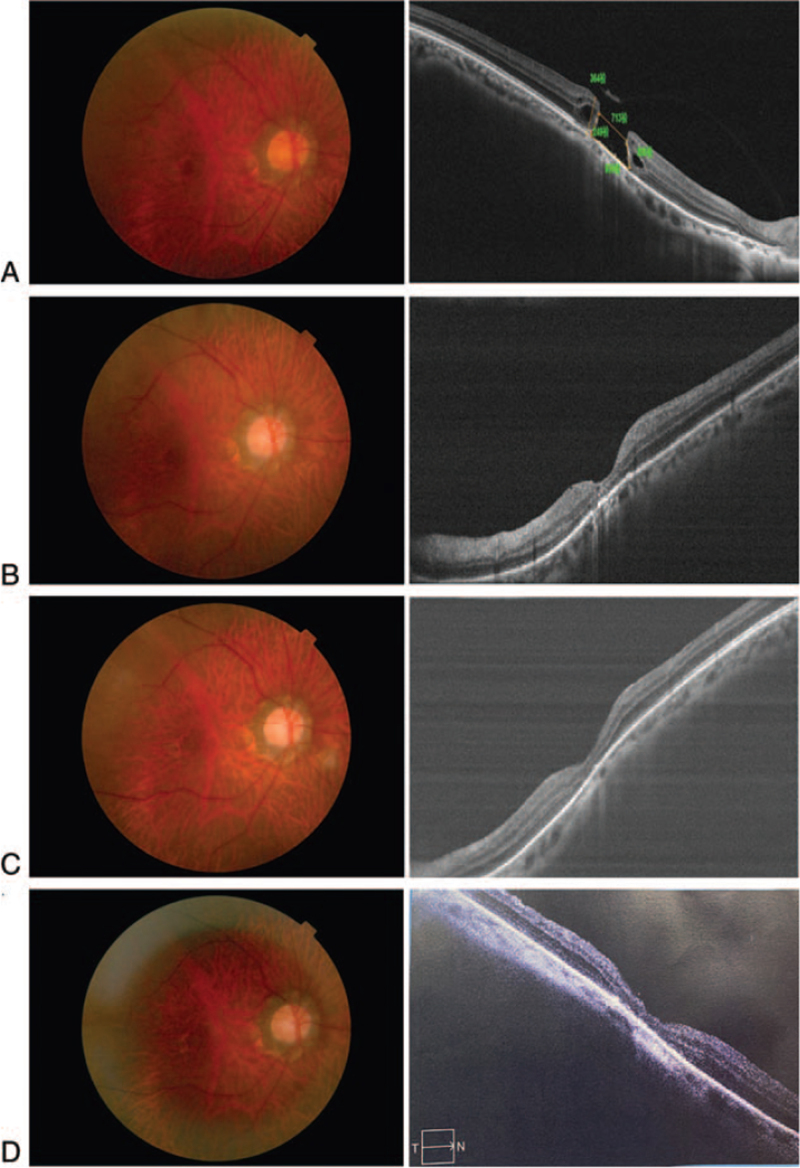
Case 2 (A) The fundus photograph image of the right eye, OCT showed the absence of full-thickness neuroepithelial tissue in the central fovea with a minimum MH diameter of 713 μm and a basal diameter of 939 μm; (B) One month after surgery, fundus image and OCT image demonstrated that the hole has closed. (C,D) Three and 6 months after surgery, the fundus image and the OCT image showed that an integral macular structure.

A 68-year-old male complained of decreased vision in the right eye for 8 months. He had no previous history of chronic diseases but had high myopia of the right eye since childhood. The BCVA (LogMAR) was 1.22 in the right eye and 0.22 in the left eye, respectively. The patient had lenticular opacity in both eyes, and the remaining anterior segments were structurally normal. Fundus examination of the right eye showed “leopard-print” changes in the retina, with posterior scleral staphyloma and visible myopic arc around the optic disc. In addition, a round hole was observed in the macular area, and vitreomacular traction was evident on the surface. SD-OCT showed the absence of full-thickness neuroepithelial tissue in the central fovea, with a minimum MH diameter of 713 μm and a basal diameter of 939 μm (Fig. 3A). Then, the patient underwent “Sandwich-type” modified ILM flap covering technique under general anesthesia in our hospital on July 15, 2020. During the surgery, a 23 G 3-incision PPV was performed to remove the vitreous in the visual axis area. Artificial PVD and excision were facilitated by triamcinolone acetonide (TA) staining. The other steps were identical to those used in Case 1. At 30 days after the surgery, complete MH closure was observed on SD-OCT and fundus imaging (Fig. 3B). Approximately 3 and 6 months after the surgery, the vision recovered to 0.92, and OCT showed MH closure (Fig. 3C,D). The patient was very satisfied with the postoperative effects.

## 3 Discussion

Although vitrectomy combined with ILM peeling has achieved a high rate of MH closure, there are still some refractory MHs with poor prognosis, for instance large MH, high myopia MH, and MH secondary to fundus diseases such as retinal detachment.^[14]^ The improvement of treatment, postoperative effects, and prognosis are yet to be intensively researched. The large chronic MH secondary to retinal detachment in Case 1 might be correlated with vitrectomy operation, residual vitreomacular traction, glial cell migration and proliferation, retinal scar traction, insufficient filling, or irregular prone position.^[15–17]^ The cause of MH secondary to high myopia might be attributed to axial elongation and posterior scleral staphyloma formation, retinal pigment epithelium, and choroid atrophy, which promote the formation of MH.^[5,18]^

The closure rate and visual outcome of full-thickness MH depend on the diameter of the MH, chronicity, state of the retinal fundus, and duration of the hole.^[19]^ The success rate of large chronic MH is low, with anatomical closure rates of 46.7% to 68.9%. The closure rate of secondary MH with severe myopia or fundus surgery is even lower.^[20,21]^ However, expanding the peeling range of the ILM flap to maximally reduce the horizontal MH traction is not an ideal approach. Therefore, in recent years, clinicians have made various modifications to ILM techniques. Some studies have shown that the ILM flap insertion technique can increase the closure rate of large MHs. However, if it is inserted insufficiently, the flap may be freed; if it is inserted excessively, scar or “mountain-shaped” extrusion may be formed. Irregular ILM clumping/contraction and excessive tissue may prevent postoperative retinal layer connection and even affect the recovery of microvascular blood flow, contributing to unsatisfactory visual improvement.^[22,23]^ The conventional single inverted ILM flap technique is effective for large MHs, but ILM flap dislocation might be problematic.^[24]^ Nonetheless, the inverted ILM flap technique has more advantages than inserted ILM flap technique in treating large-diameter MH, as it may act as a “bridge” similarly to the basement membrane, which provides a scaffold for glial cells proliferation. At the same time, the inverted ILM flap, which isolates the vitreous cavity from MH, reduces the interference of subsequent surgical operations on the retinal pigment epithelium. It also forms a relatively stable internal environment and promotes MH.^[24,25]^ Presently, heavy water is often used to fix the inverted ILM in the clinic, but the toxic effects on the retina cannot be ignored. Therefore, we adopted the “Sandwich-type technique.” Autologous blood was dripped into the MH and then stained with ICG, as it contains various growth factors and collagen, which promotes the proliferation and migration of cells and plays an important role in the healing of super-large MHs. It also reduces the toxic effect of ICG while enhancing the adhesion between the retinal pigment epithelium and the neuroepithelial layer.^[26]^ The 270°C-shaped temporal ILM flap was peeled and covered the MH at a distance of 0.5 DD from the foveal center. This covering method fully released the ILM traction, reduced the tension at the edge of the hole, and perfectly preserved the function of the nasal maculopapillary nerve fiber bundle, which has critical foveal conduction between the fovea and optic nerve. In addition, the ILM flap of 1.5 diameter size disc avoided curling and folding when the flap was covered, and the foveal contour could also be well restored at this peeling size. Finally, autologous blood was dripped to seal the flap and prevent it from shifting during fluid-air exchange to avoid retinal toxicity caused by heavy water. The postoperative visual acuities of two patients with large chronic MHs improved to varying degrees, and the integrity of the macular structure recovered well. It can be concluded that “Sandwich-type” modified ILM flap covering technique makes up for the deficiency of conventional single-inverted ILM flap covering technique, and takes advantage of growth factors of autologous blood that promote the proliferation, migration of glial cells, and the closure of MH.

In conclusion, our case report suggests that the “Sandwich-type ILM flap covering technique can be an effective treatment for large chronic MH caused by high myopia or retinal detachment.” However, future randomized trials may be helpful in determining the long-term effects and risks of this method in treating large chronic MH. In addition, these results suggest the need to perform further studies to identify the presence of risk factors that could increase the probability of failure.

## Acknowledgments

We thank the Affiliated Eye Hospital of Nanjing Medical University for supporting this research.

## Author contributions

KL and YZ carried out the studies, participated in data collection, and drafted the manuscript. WY and QJ participated in the study design. XX performed the surgery. All authors read and approved the final manuscript.

**Data curation:** Keran Li, Yunfan Zhou.

**Investigation:** Xiangzhong Xu.

**Methodology:** Weihua Yang, Qin Jiang.

**Writing – original draft:** Keran Li, Yunfan Zhou.

## References

[1] Dai Y Dong F Zhang X Yang Z. Internal limiting membrane transplantation for unclosed and large macular holes. Graefes Arch Clin Exp Ophthalmol 2016;254:2095–9. 27520464 10.1007/s00417-016-3461-4

[2] Gross JG. Late reopening and spontaneous closure of previously repaired macular holes. Am J Ophthalmol 2005;140:556–8. 16139019 10.1016/j.ajo.2005.03.044

[3] Morescalchi F Costagliola C Gambicorti E Duse S Romano MR Semeraro F. Controversies over the role of internal limiting membrane peeling during vitrectomy in macular hole surgery. Surv Ophthalmol 2017;62:58–69. 27491476 10.1016/j.survophthal.2016.07.003

[4] Pichi F Abboud EB. Spare some internal limiting membrane for later: free ILM patch and neurosensory retina graft. Int Ophthalmol 2019;39:1205–7. 29637388 10.1007/s10792-018-0906-2

[5] Ortisi E Avitabile T Bonfiglio V. Surgical management of retinal detachment because of macular hole in highly myopic eyes. Retina 2012;32:1704–18. 23007668 10.1097/IAE.0b013e31826b671c

[6] Sabry D El-Kannishy A Kamel R Abou Samra W. Correlation between en face optical coherence tomography defects of the inner retinal layers and ganglion cell inner plexiform layer analysis after internal limiting membrane peeling for idiopathic full-thickness macular hole. Invest Ophthalmol Vis Sci 2016;57:444–50. 27409504 10.1167/iovs.15-18043

[7] Almony A Nudleman E Shah GK. Techniques, rationale, and outcomes of internal limiting membrane peeling. Retina 2012;32:877–91. 22105502 10.1097/IAE.0b013e318227ab39

[8] Tognetto D Grandin R Sanguinetti G. Internal limiting membrane removal during macular hole surgery: results of a multicenter retrospective study. Ophthalmology 2006;113:1401–10. 16877079 10.1016/j.ophtha.2006.02.061

[9] Shpak AA Shkvorchenko DO Sharafetdinov I Yukhanova OA. Predicting anatomical results of surgical treatment of idiopathic macular hole. Int J Ophthalmol 2016;9:253–7. 26949645 10.18240/ijo.2016.02.13 PMC4761737

[10] Miller JB Yonekawa Y Eliott D. Long-term follow-up and outcomes in traumatic macular holes. Am J Ophthalmol 2015;160:1255–8.e1251. 26393438 10.1016/j.ajo.2015.09.004

[11] Azzolini C. Macular hole: from diagnosis to therapy. J Ophthalmol 2020;1473763. 32280514 10.1155/2020/1473763 PMC7125507

[12] Yu Y Liang X Wang Z Wang J Liu W. Clinical and morphological comparisons of idiopathic macular holes between stage 3 and stage 4. Graefes Arch Clin Exp Ophthalmol 2018;256:2327–33. 30315410 10.1007/s00417-018-4158-7

[13] Hu Z Lin H Liang Q Wu R. Comparing the inverted internal limiting membrane flap with autologous blood technique to internal limiting membrane insertion for the repair of refractory macular hole. Int Ophthalmol 2020;40:141–9. 31463622 10.1007/s10792-019-01162-0

[14] De Novelli FJ Preti RC Ribeiro Monteiro ML, et al. Autologous internal limiting membrane fragment transplantation for large, chronic, and refractory macular holes. Ophthalmic Res 2015;55:45–52. 26569390 10.1159/000440767

[15] Lee SH Park KH Kim JH. Secondary macular hole formation after vitrectomy. Retina 2010;30:1072–7. 20168267 10.1097/IAE.0b013e3181cd4819

[16] Brown GC. Macular hole following rhegmatogenous retinal detachment repair. Arch Ophthalmol 1988;106:765–6. 3370002 10.1001/archopht.1988.01060130835034

[17] Shibata M Oshitari T Kajita F, et al. Development of macular holes after rhegmatogenous retinal detachment repair in Japanese patients. J Ophthalmol 2012;2012(740591. 10.1155/2012/740591 PMC3270438 22315662

[18] Wei Y Wang N Zu Z. Efficacy of vitrectomy with triamcinolone assistance versus internal limiting membrane peeling for highly myopic macular hole retinal detachment. Retina 2013;33:1151–7. 23508079 10.1097/IAE.0b013e31827b6422

[19] Salter AB Folgar FA Weissbrot J Wald KJ. Macular hole surgery prognostic success rates based on macular hole size. Ophthalmic Surg Lasers Imaging 2012;43:184–9. 22320413 10.3928/15428877-20120102-05

[20] Stec LA Ross RD Williams GA, et al. Vitrectomy for chronic macular holes. Retina 2004;24:341–7. 15187653 10.1097/00006982-200406000-00001

[21] Charles S Randolph JC Neekhra A, et al. Arcuate retinotomy for the repair of large macular holes. Ophthalmic Surg Lasers Imaging Retina 2013;44:69–72. 23418735 10.3928/23258160-20121221-15

[22] Iwasaki M Kinoshita T Miyamoto H Imaizumi H. Influence of inverted internal limiting membrane flap technique on the outer retinal layer structures after a large macular hole surgery. Retina 2019;39:1470–7. 29863535 10.1097/IAE.0000000000002209

[23] Park JH Lee SM Park SW, et al. Comparative analysis of large macular hole surgery using an internal limiting membrane insertion versus inverted flap technique. Br J Ophthalmol 2019;103:245–50. 29610221 10.1136/bjophthalmol-2017-311770

[24] Xu Q Luan J. Internal limiting membrane flap technique in macular hole surgery. Int J Ophthalmol 2020;13:822–31. 32420232 10.18240/ijo.2020.05.19 PMC7201361

[25] Faria MY Proença H Ferreira NG, et al. Inverted interbal limiting membrane flap techniques and outer retinal layer structures. Retina 2020;40:1299–305. 31259810 10.1097/IAE.0000000000002607

[26] Lyu WJ Ji LB Xiao Y, et al. Treatment of refractory giant macular hole by vitrectomy with internal limiting membrane transplantation and autologous blood. Int J Ophthalmol 2018;11:818–22. 29862182 10.18240/ijo.2018.05.17 PMC5957035

